# Novel genetic alteration in congenital melanocytic nevus: MAP2K1 germline mutation with BRAF somatic mutation

**DOI:** 10.1186/s41065-020-00147-9

**Published:** 2020-08-26

**Authors:** Yun Zou, Yi Sun, Xiaojing Zeng, Yun Liu, Qingqing Cen, Hao Gu, Xiaoxi Lin, Ren Cai, Hui Chen

**Affiliations:** 1 Department of Plastic and Reconstructive Surgery, Shanghai 9th Peoples Hospital Affiliated to Shanghai Jiaotong University, Shanghai, China; 2 Children’s Hospital, Zhejiang University School of Medicine, National Clinical Research Center for Child Health, National Children’s Regional Medical Center, Hangzhou, China; 3 Institutes of Biomedical Sciences, Fudan University, Shanghai, PR China; 4 Bio-X Institute, Shanghai Jiao Tong University, Shanghai, China

## Abstract

Congenital melanocytic nevus (CMN) represent a benign proliferative skin disease in the epidermis and dermis. CMN are historically known to be associated with activating NRAS or BRAF mutations. Melanoma frequently harbors the BRAF p.Val600Glu mutation, which is also commonly found in benign nevi. A recent study reported mutation of MAP2K1, a downstream effector of the RAS-RAF-MEK pathway, in melanoma with an overall frequency of 8%. Later, in 2019, Jansen P detected one activating MAP2K1 mutation in acral nevi. However, it is unknown whether MAP2K1 mutations are common in CMN, and how MAP2K1 contributes to the pathogenesis of CMN remains to be determined.

In this study, we report one patient clinically and histologically diagnosed with CMN, with the MAP2K1 germline mutation and a BRAF p.Val600Glu somatic hit in the lesion. To the best of our knowledge, this is the first report of the coexistence of mutated BRAF and MAP2K1 in CMN, which may suggest that MAP2K1 mutations contribute to the occurrence and development of nevus expanding our knowledge of the genetics of CMN.

## Background

Congenital melanocytic nevus (CMN) represent a benign proliferative skin disease in the epidermis and dermis that presents at birth or in the first few weeks of life and affects approximately 1–2% of newborns [1]. CMN can present as dark pigmented lesions with rugose, pebbly, verrucous, or even cerebriform surfaces [2]. Nevi lesions may become progressively darker and thicker with age. Although biologically considered benign diseases, it has been confirmed that CMN can be a precursor of melanoma given their shared common driver mutations [3, 4]. CMN are historically known to be associated with activating NRAS mutations. According to the literature, melanoma, a malignant cancer, frequently harbors the BRAF p.Val600Glu mutation [5]. In addition, BRAF p.Val600Glu was also identified in CMN [6], suggesting that the alteration constitutes an early key somatic event in the transformation into melanoma. Nikolaev et al. detected mutation of MAP2K1, a downstream effector in the RAS-RAF-MEK pathway, in melanoma with an overall frequency of 8% [7]. Later, in 2019, Jansen P detected one activating MAP2K1 mutation in acral nevi [8]. However, it is unknown whether MAP2K1 mutations are common in CMN, and how MAP2K1 contributes to the pathogenesis of CMN remains to be determined.

In this study, we report one patient clinically and histologically diagnosed with CMN with a MAP2K1 germline mutation and a BRAF p.Val600Glu somatic mutation in the same lesion. A novel MAP2K1 mutation was detected in CMN for the first time, which may suggest that the MAP2K1 mutation contributes to the occurrence and development of nevus, expanding our knowledge of the genetics of CMN.

## Methods

This study was conducted following principles in the Declaration of Helsinki and was approved by the Ethics Committee of Shanghai Ninth People’s Hospital Affiliated to Shanghai Jiaotong University School of Medicine (equivalent to the Institutional Review Board). Verbal and written informed consent for study participation and publication of clinical images and identifying information was granted prior to the study. Clinical data, including detailed medical history, physical examination, histological examination and molecular analysis results, were recorded.

## Case presentation

**Fig. 1:**
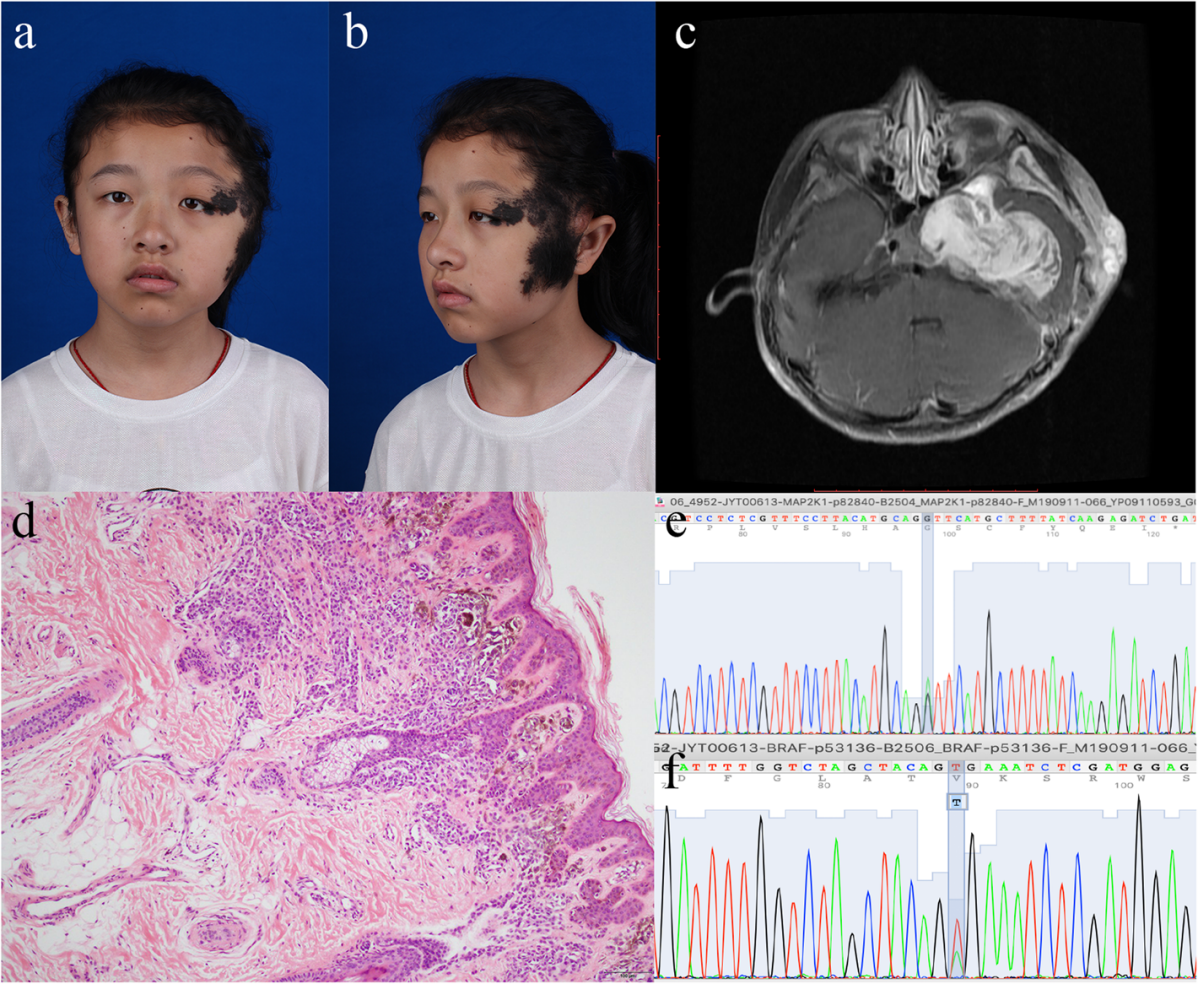
**a**-**b** Clinical manifestation of the patient: left-sided swelling and congenital melanocytic nevus on the left temporal area. The lesion was flat with irregular boundary and hypertrichosis. **c** T2-weighted post-contrast magnetic resonance imaging (MRI) axial view demonstrated heterogeneous enhancement of fossa cranii media, basis cranii, and temporal area on the right, as well as compression and displacement of left temporal lobe, consist with the diagnosis of vascular malformation. **d** The infiltrated melanocyte in the epidermis and dermis in the microscopic study (hematoxylin–eosin, original magnification × 100). **e** Sanger sequence of germline mutation of MAP2K1(15:66782840 G > A). **f** Sanger sequence of somatic mutation of BRAF (7:140453136 A > T)

A 12-year-old female patient presented with left-sided swelling and congenital melanocytic nevus on the left temporal area with an unremarkable family history (Fig. 1a, b). The lesion was flat with irregular boundaries and hypertrichosis. A medical history of intracranial and extracranial venous malformations and severe headache for 4 years was noted. T1-weighted postcontrast MR imaging demonstrated heterogeneous enhancement of the fossa cranii media, basis cranii, and temporal area on the right, as well as compression and displacement of the left temporal lobe (Fig. 1c).

Because of the deep location of the vascular lesions and the connectedness of the intracranial and extracranial lesions, we performed minimally invasive biopsy on the nevus in the hair line (Fig. 1) (D = 2 mm, all layer biopsy) under local anesthesia (Penles and 1% xylocaine) to determine the histological and genetic changes. In addition to tissue specimens, blood specimens from peripheral veins were also collected. One tissue specimen was fixed in 10% formaldehyde solution, embedded in paraffin, sectioned, and stained with hematoxylin-eosin for histopathological analysis. Pathology analysis was performed by two qualified and experienced pathologists. Next-generation sequencing (NGS) was performed with tissue as well as peripheral blood.

### Next-generation sequencing (NGS)

A targeted NGS panel was designed with genes related to CMN (NRAS, BRAF, KRAS, HRAS, and MAP2K1). SureSelect XT kit reagents (Agilent Technologies, Santa Clara, CA) were used for the Illumina adapters. Quantitative PCR (KAPA Biosystems, Willmington, MA) was used to verify the concentration of the indexed sample, and then the sample was sequenced on a MiSeq instrument (Illumina, San Diego, CA) using 150 paired-end reads. By using the Integrative Genomic Viewer, variants were analyzed.

### Sanger sequencing

The MAP2K1 and BRAF genes were PCR-amplified to track the 15:66782840 G > A, c.1069G > A, p.Val357Ile, and 7:140453136 A > T, c.1799 T > A, p.Val600Glu mutations through the blood and tissue samples. Primer sequences are available upon request. Amplicon fragments were sequenced bidirectionally (forward and reverse) with M13 primer with the Big Dye1 Terminator v3.1 cycle sequencing kit and an ABI 3730 DNA Analyzer (Life Technologies, Carlsbad, CA). Target sequences were compared to the MAP2K1 and BRAF reference sequences using Mutation Surveyor (SoftGenetics, State College, PA).

## Result

Histology showed infiltrating melanocytes in the epidermis and dermis (Fig. 1d).

The genetic study identified a germline mutation in MAP2K1 (p.Val357lle; SIFT score, 0.19; PolyPhen-2-HDIV score, 0.001; PolyPhen-2-HAVR score, 0.008) in the blood at a mutation frequency of 48.51% (at a depth of 5850x), which was detected by Sanger sequencing (Fig. 1e). In addition to the germline mutation, a somatic mutation in the gene BRAF (p.Val600Glu) was detected in tissue specimens at a frequency of 11.86% (at a depth of 6221x, Fig. 1f).

## Discussion

Congenital melanocytic nevus (CMN), although with rare reports of familial cases, is overwhelmingly considered to occur in a sporadic pattern. According to the literature, NRAS mutations and, to a lesser extent, BRAF mutations are the most common genetic hits underlying CMN pathogenesis [9–12]. In melanoma, NRAS and BRAF mutations have also been detected and used as genetic markers in tools integrating genetic and morphologic features to improve classification [4]. NRAS, one of the three major isoforms of the RAS family, is involved in cell growth, differentiation, and survival. BRAF, a serine–threonine kinase, is activated by the RAS family. Activating BRAF can trigger the MAPK signaling pathway, ultimately leading to cell cycle progression, transcription upregulation, and differentiation. The high frequency of oncogenic BRAF V600E mutation reported in melanoma and melanocytic nevus suggests that activation of the RAS/RAF/MEK/ERK pathway is a critical step in the development of melanocytic diseases.

Current genomic studies of benign melanocytic lesions targeting genes classically involved in melanoma genesis have resulted in a lack of comprehensive molecular profiling of CMN, in contrast to the genome-wide studies of melanomas. Colebatch AJ et al. [13] found that all nevi had driver mutations in in the MAPK signaling pathway in the form of either BRAF V600E or NRAS Q61R/L by performing whole-genome sequencing on 14 benign melanocytic nevi. In 2012, Nikolaev et al. performed exome sequencing to detect somatic mutations in melanoma and found the presence of somatic mutations in MAP2K1/MAP2K2, downstream effectors of the RAS-RAF-MEK pathway, with an overall frequency of 8%. Moreover, the coexistence of mutated BRAF and gain-of-function MAP2K1/MAP2K2 mutations was detected in two melanomas and resulted in higher resistance to MEK inhibitors [7]. Recently, activating MAP2K1 mutations has also been detected in benign nevi. In 2019, Jansen P reported an activating MAP2K1 mutation at an overall frequency of 1% in acral nevi [8]. Later in 2020, Muthiah S et al. reported MAP2K1 (c.607G > A, p.E203K) in naevus spilus (NS)-type CMN [14].However, it is still unknown whether MAP2K1 mutations are characteristic of CMN, and how MAP2K1 contributes to the pathogenesis of CMN remains to be determined.

The coexistence of vascular anomalies and CMN is rare but important. In 2018, Etchevers H. C. et al. [15] detected a somatic BRAF mutation in a patient diagnosed with giant CMN associated with vascular malformation and epidermal cysts. The biopsy demonstrated venous malformation intermingled with the CMN. In our patient, the vascular malformation and CMN lesion are located in different layers of tissue structure in the same site. MAP2K1, the downstream effector of BRAF, has been wildly identified in extracranial and intracranial AVMs [16–18]. RASopathy, a group of disorders affecting the RAS-MAPK signal pathway, include Noonan syndrome, cardiofaciocutaneous syndrome, Costello syndrome, neurofibromatosis type 1 [19, 20]. The patient did not fulfill criteria for RASopathy based on the absence of distinctive facial features, abnormal genitalia, growth retardation and ECG abnormalities. In this study, we only preformed tissue biopsy in the superficial lesion due to the biopsy of deep vascular malformation lesions may be difficult and lead to uncontrollable bleeding. Although it is difficult to explain the coexistence of vascular anomalies and CMN, the RAS-RAF-MEK pathway can affect both pigment and endothelial cell physiology [4, 18], which raises up our awareness of these potentially important biologic phenomenon in further investigation. We hypothesize that a postzygotic mutation during development may account for this clinical presentation.

Genetic studies provide information for a comprehensive understanding of CMN, and molecular targeted therapies also provide new management strategies, especially for large to giant CMN, since traditional treatment relies heavily on surgical excision procedures. In 2019, Mir A et al. [21] reported the first targeted therapy in a *BRAF*-mutated giant CMN with trametinib, a MEK inhibitor. Later, Rouille et al. [22] reported that the proliferative potential of CMN could be limited by local administration of MEK and Akt inhibitors in a xenograft model.

In this study, the patient was diagnosed with CMN based on clinical manifestations and histology results according to the diagnostic criteria. This is the first reported case of CMN with a MAP2K1 germline mutation and a BRAF (p.Val600Glu) somatic hit. It is still unclear whether the MAP2K1 mutation occurs in an early stage of melanocytic development. These questions, as well as the potential functional impact of combinations of MAP2K1 and BRAF mutations, will have to be further addressed. More notably, the potential application of molecular targeted drugs provides a new strategy for the treatment of giant CMN.

## Conclusion

In this study, we report one patient clinically and histologically diagnosed with CMN, with the MAP2K1 germline mutation and a BRAF (p.Val600Glu) somatic hit in the lesion. To the best of our knowledge, this is the first description of the coexistence of mutated BRAF and MAP2K1 in CMN. Novel genetic mutations in CMN beyond BRAF and NRAS mutations in the MAPK signaling pathway were identified. Such information will help us understand the pathogenesis of CMN and obtain comprehensive molecular profiling in CMN.

## Data Availability

All data used during the study appear in the submitted article.

## Abbreviations

CMN: Congenital melanocytic nevus
NGS: Next-generation sequencing
